# A generic emergency protocol for patients with inborn errors of metabolism causing fasting intolerance: A retrospective, single‐center study and the generation of www.emergencyprotocol.net

**DOI:** 10.1002/jimd.12386

**Published:** 2021-05-04

**Authors:** Alessandro Rossi, Irene J. Hoogeveen, Charlotte M. A. Lubout, Foekje de Boer, Marieke J. Fokkert‐Wilts, Iris L. Rodenburg, Esther van Dam, Sarah C. Grünert, Diego Martinelli, Maurizio Scarpa, CONNECT MetabERN Collaboration Group, Hanka Dekker, Sebastiaan T. te Boekhorst, Francjan J. van Spronsen, Terry G. J. Derks

**Affiliations:** ^1^ Section of Metabolic Diseases Beatrix Children's Hospital, University Medical Centre Groningen, University of Groningen Groningen The Netherlands; ^2^ Department of Translational Medicine, Section of Pediatrics University of Naples "Federico II" Naples Italy; ^3^ Department of General Pediatrics, Adolescent Medicine and Neonatology Medical Centre‐University of Freiburg, Faculty of Medicine Freiburg Germany; ^4^ Division of Metabolism, Bambino Gesù Children's Hospital IRCCS Rome Italy; ^5^ metabERN, Regional Center for Rare Diseases University Hospital of Udine Udine Italy; ^6^ VKS, The Dutch Patient Association for Inherited Metabolic Diseases Zwolle The Netherlands; ^7^ Patient Connect B.V Bilthoven The Netherlands

**Keywords:** eHealth, emergency treatment, fatty acid oxidation disorders, glycogen storage diseases, hypoglycemia, telemedicine

## Abstract

Patients with inborn errors of metabolism causing fasting intolerance can experience acute metabolic decompensations. Long‐term data on outcomes using emergency letters are lacking. This is a retrospective, observational, single‐center study of the use of emergency letters based on a generic emergency protocol in patients with hepatic glycogen storage diseases (GSD) or fatty acid oxidation disorders (FAOD). Data on hospital admissions, initial laboratory results, and serious adverse events were collected. Subsequently, the website www.emergencyprotocol.net was generated in the context of the CONNECT MetabERN eHealth project following multiple meetings, protocol revisions, and translations. Representing 470 emergency protocol years, 127 hospital admissions were documented in 54/128 (42%) patients who made use of emergency letters generated based on the generic emergency protocol. Hypoglycemia (here defined as glucose concentration < 3.9 mmol/L) was reported in only 15% of hospital admissions and was uncommon in patients with ketotic GSD and patients with FAOD aged >5 years. Convulsions, coma, or death was not documented. By providing basic information, emergency letters for individual patients with hepatic GSD or the main FAOD can be generated at www.emergencyprotocol.net, in nine different languages. Generic emergency protocols are safe and easy for home management by the caregivers and the first hour in‐hospital management to prevent metabolic emergencies in patients with hepatic GSD and medium‐chain Acyl CoA dehydrogenase deficiency. The website www.emergencyprotocol.net is designed to support families and healthcare providers to generate personalized emergency letters for patients with hepatic GSD and the main FAOD.

**Abbreviations**
CK: creatine kinase
EHR: electronic health record
FAOD: fatty acid oxidation defects
GSD: glycogen storage disease
ICU: intensive care unit
IEM: inborn error of metabolism
LCHADD: long‐chain 3‐hydroxyacyl‐CoA dehydrogenase deficiency
MADD: multiple‐chain Acyl CoA dehydrogenase deficiency
MCADD: medium‐chain Acyl CoA dehydrogenase deficiency
VLCADD: very long‐chain Acyl CoA dehydrogenase deficiency

## 1 INTRODUCTION

Fasting intolerance is a critical feature of several rare inborn errors of metabolism (IEMs), including hepatic glycogen storage diseases (GSD) and fatty acid oxidation disorders (FAOD). If inadequately treated or not prevented, fasting intolerance can lead to acute life‐threatening complications, such as (severe) hypoglycemia; metabolic acidosis; and eventually convulsions, coma, or death. For these reasons, it is crucially important that following the diagnosis of such IEMs, metabolic decompensations and emergencies are effectively prevented, risk situations are recognized in a timely fashion, and that prompt safe treatment is established rapidly to stop and reverse catabolism.^1^ Catabolism is a key trigger of clinical and metabolic decompensation and is often induced by (the combination of) fever, prolonged fasting (eg, decreased oral/enteral intake due to illnesses, or surgery‐related fasting protocols), increased enteral losses resulting from vomiting and/or diarrhea, or alcohol excess. Therefore, the key initial measure in IEM emergency protocols is to stop catabolism and promote anabolism.^2^, ^3^, ^4^

Information about IEM‐specific emergency protocols is available at multiple online resources, such as the New England Consortium of Metabolic Programs, British Inherited Metabolic Diseases group (https://www.newenglandconsortium.org/acute-illness), and INVEST (in Dutch: Internisten voor volwassenen met een erfelijke stofwisselingsziekte) (https://investof.nl/noodprotocollen/), and scientific publications, such as for urea cycle defects,^5^ maple syrup urine disease,^6^ organic acidemias,^7^ FAOD,^8^ or incorporated in guidelines for glutaric aciduria type I^9^ and subtypes of hepatic GSD.^10^, ^11^, ^12^, ^13^, ^14^ These guidelines and emergency protocols are largely based on expert opinions. Follow‐up studies are not available on practices of emergency treatments.

The question “How should sickness and emergency situations be managed for patients with liver GSD?” has been recently ranked as a top priority for research in the international priority setting partnership for liver GSD.^15^ Given the geographical distance between centers of expertise and the home address of patients with IEMs, local or regional healthcare providers are often the ones starting the initial emergency treatment. However, it is recognized that most (pediatrics) residents/physicians consider they have insufficient knowledge to start emergency treatment for patients with IEMs in the absence of expert advice or written protocols.^16^ Finally, keeping emergency letters up to date for large cohorts of patients with IEMs can be labor intensive.

The aim of this report is 2‐fold. First, we describe a retrospective, observational, single‐center study about the application of emergency letters based on a simple *generic* emergency protocol for patients with IEMs causing fasting intolerance. Second, we report the development of the website www.emergencyprotocol.net, where personalized emergency letters can be automatically created for and by (families and) patients with FAOD and hepatic GSD.

## 2 METHODS

### 2.1 Ethics

For the retrospective, observational, single‐center medical chart review in patients with hepatic GSD or FAOD, the Medical Ethical Committee of the University Medical Center Groningen (UMCG) stated that the Medical Research Involving Human Subjects Act was not applicable. Official study approval by the Medical Ethical Committee was not required (METc 2019/119) because the study involved retrospective, anonymous data collection of standardized care.

### 2.2 The UMCG generic emergency letters

**FIGURE 1:**
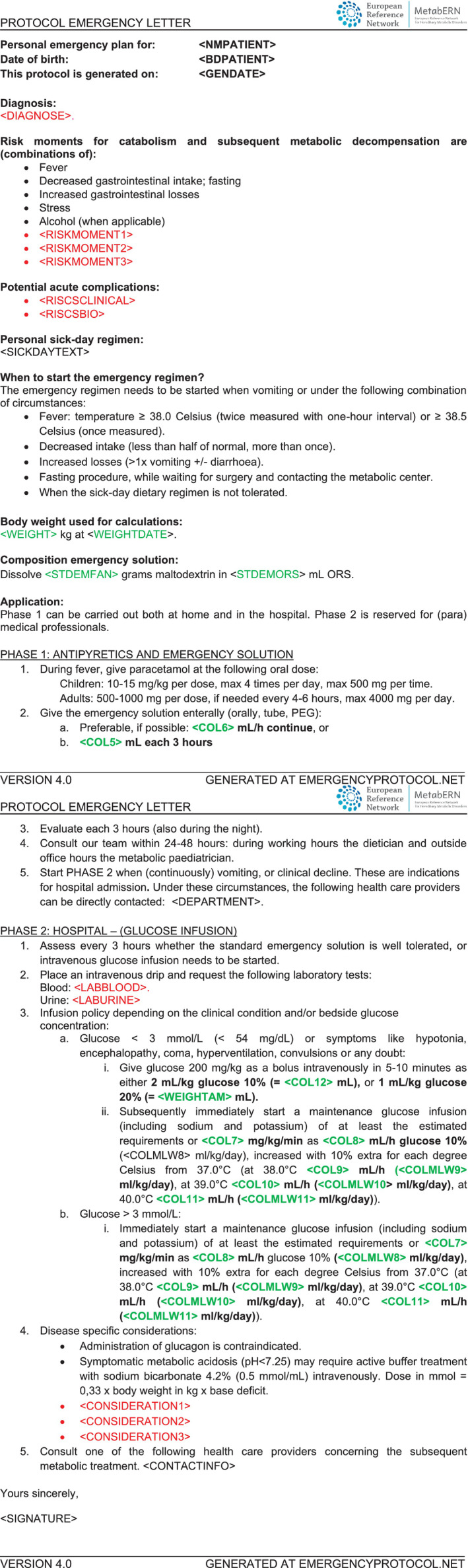
“Emergency protocol for children at risk for acute metabolic decompensation” template. Variables depending on patients' body weight are shown in blue; variables depending on the specific IEM are shown in green

Since February 2014, individualized, IEM‐specific emergency letters have been replaced by emergency letters based on the *generic* “Emergency protocol for children at risk for acute metabolic decompensation” at UMCG (Figure 1). Patients (and their families) have been instructed at the outpatient clinic and in hospital about the prevention of catabolism, how to use the emergency letter, and how to directly seek for healthcare professional support during acute hospital admissions. In brief, the protocol includes two phases. Phase I can be initiated by caregivers or patients at home under the following circumstances: (1) more than one‐time vomiting, or (2) a combination of (a) fever >38.5°C, (b) decreased enteral intake, and (c) increased enteral losses. Phase I prescribes (a) a weight‐dependent dose of paracetamol (acetaminophen) to reduce fever, and (b) the administration of the “emergency solution” to provide enough carbohydrates.

Before 2014, several patients with IEMs at UMCG had reported emergency treatments in local hospitals, which were complicated by hypoglycemia after administration of oral rehydration salt solutions (relatively low in calories and thus unable to stop or reverse catabolism). Therefore, in our generic protocol, we have ensured that total fluid maintenance requirements per 24 hours include glucose polymer enrichment, as described by Van Hove et al,^4^ with slight simplifications. In this so‐called emergency solution, total carbohydrate prescriptions are based on experimental data on carbohydrate requirements using stable isotopes.^17^ The emergency solution provides, in 500 mL of oral rehydration salt solution (ORS), 75 g of maltodextrin (15 g per 100 mL of solution) for patients weighing up to 12 kg and 110 g (20 g per 100 mL of solution) for patients weighing 12 kg or more, respectively. A stand‐alone product is currently lacking. Therefore, in the Netherlands, the maltodextrin is currently provided by the metabolic dietitian through a facility company, while the ORS can be purchased in local drugstores. The protocol is updated when the body weight changes more than 10%. If phase I is not tolerated or ineffective, the protocol moves to phase II. For phase II, local physicians (pediatricians, internal medicine specialists) are asked to provide patients with direct access to the emergency or general department to ensure prompt enteral or parenteral carbohydrate administration. The protocol advises physicians to contact the metabolic consultant on call when the initial laboratory results are available, usually within 1 hour after hospital admission. At this point, the generic approach and emergency letter treatment change into personalized management plan, based on the specific IEM and patient.

### 2.3 Subjects

Clinical and laboratory data from emergency department visits and hospital admissions were retrieved from the electronic health record (EHR) system of the UMCG for the period 1 February 2014 to 24 April 2019. Inclusion criteria were a confirmed diagnosis of hepatic GSD or FAOD, and the presence of an emergency letter based on the *generic* emergency protocol. Patients for whom UMCG was not the primary responsible center in the entire healthcare chain were excluded. Patients were classified as children (age < 16 years) or adults (age ≥ 16 years). Data were abstracted on the number of admissions due to a metabolic emergency, the percentage of patients with hypoglycemia at admissions, and the occurrence of serious adverse events (defined as intensive care unit [ICU] admission, coma, or death). Neurological symptoms (convulsions, lethargy) and blood concentrations of creatine kinase (CK) and ammonia were also recorded.

### 2.4 Data analysis

Study data were collected and managed using REDCap electronic data capture tools hosted at the UMCG.^18^ Hypoglycemia was conservatively defined as blood glucose concentrations <3.9 mmol/L, based on glycemic thresholds for activation of counterregulatory systems.^19^

### 2.5 Generation of www.emergencyprotocol.net

This project aimed at being as inclusive as possible. Invitations were sent to healthcare professionals and patient organization representatives after the society for the study of inborn errors of metabolism Sponsored Satellite Symposium “Emergency regimes: current status and options for improvement”, the European Metabolic Group meeting Workshop “Dietary management in GSD type I", and the IGSD2017 Networking session “Emergency protocols for hepatic GSD”. Additional contribution came from (national/international) patient organizations meetings.

In the CONNECT MetabERN eHealth project, activity 3 was focused on the automatical generation of emergency letters for patients with GSD and FAOD. After initial meeting in Hannover on 2 December 2019, the UMCG emergency protocol has been revised by multiple healthcare providers during discussions in online meetings on 27 February 2020 and 1 April 2020, input by emails, and via a SurveyMonkey questionnaire (sent on 20 March 2020; 36 responses).

After agreement on the English template to generate emergency letters, since 15 April 2020, the website www.emergencyprotocol.net has been designed and published on 23 June 2020 during a webinar for families and healthcare providers. Meanwhile, translations have been created for the patient information leaflets (providing instructions on how to use the emergency letter) and the emergency letter templates into the following languages: Dutch, English, French, German, Greek, Italian, Polish, Portuguese, Spanish, Swedish, and Turkish. The international validity of the protocol was guaranteed by the contribution of native tongue language editors/healthcare professionals/patient organizations who are part of the CONNECT MetabERN collaboration group. After agreement among native tongue language editors for each of the abovementioned languages, the translated versions were released on the website.

## 3 RESULTS

### 3.1 Subjects

In total, 128 patients (66 males, 62 females) with hepatic GSD or an FAOD were included. Of these, 95/128 (74%) were children, 33/128 (26%) were adults. Median age at implementation of the *generic* emergency protocol was 12 years (range: 0‐50 years): <12 months (n = 10 patients); 1 to 5 years (n = 35); 6 to 10 years (n = 35); 11 to 15 years (n = 14); and >16 years (n = 34 patients), respectively. The cohort contributed a total of 470 emergency protocol years. The type and distribution of the specific IEM were as follows: medium‐chain Acyl CoA dehydrogenase deficiency (MCADD) (n = 63, 49%), hepatic GSD (n = 59, 46%), multiple‐chain Acyl CoA dehydrogenase deficiency (MADD) (n = 3, 2%), long‐chain 3‐hydroxyacyl‐CoA dehydrogenase deficiency (LCHADD) (n = 2, 2%), and very‐long‐chain Acyl CoA dehydrogenase deficiency (VLCADD) (n = 1, 1%). One patient was excluded from data analysis, because of severe medical and psychosocial comorbidities that complicated the interpretation of hospital admissions.

### 3.2 Outcomes of the application of generic emergency letters

Table 1 presents an overview of the 127 hospital admissions documented in 54 of 128 patients (42%). Patients' ages at admission were as follows: < 12 months (n = 2 admissions); 1 to 5 years (n = 71); 6 to 10 years (n = 20); 11 to 15 years (n = 7); >16 years (n = 19), respectively. Exact information on age was not available for eight admissions. Hospital admission was considered unnecessary in 11 presentations at the emergency department, representing seven individual patients.

**TABLE 1:** Overview of hospital admissions during metabolic decompensation in 128 patients with an IEM associated with fasting intolerance

IEM	Total of patients, n	Total admissions, n	Unique patients with admission, n (%)^a^	Number of patients with ≥1 admission × number of admissions	Median age^b^, years [range]
GSDIa	23	25	8 (35%)	1 × 10 1 × 5 1 × 3 2 × 2 3 × 1	18 [1‐39]
GSDIb	7	10	4 (57%)	2 × 4 2 × 1	13 [4‐19]
GSDIIIa	8	7	3 (38%)	1 × 3 2 × 2	8 [6–11]
GSDIIIb	3	0	—	—	—
GSDVI	1	0	—	—	—
GSDIX	15	16	7 (47%)	1 × 4 3 × 3 3 × 1	3 [0–6]
GSDXI	2	1	1 (50%)	1 × 1	6 [NA]
MCADD	63	50	26 (41%)	4 × 4 3 × 3 6 × 2 13 × 1	3 [0‐13]
MADD	3	14	2 (67%)	2 × 7	4 [0‐21]
LCHADD	2	3	2 (100%)	1 × 2 1 × 1	4 [0–5]
VLCADD	1	1	1 (100%)	1 × 1	4 [NA]
Total population	128	127	54 (42%)	54 × 127	8 [0‐39]

^a^ % is the number of unique patients with admission divided by total number of patients with a specific IEM.

^b^ Age at hospital admission.

**FIGURE 2:**
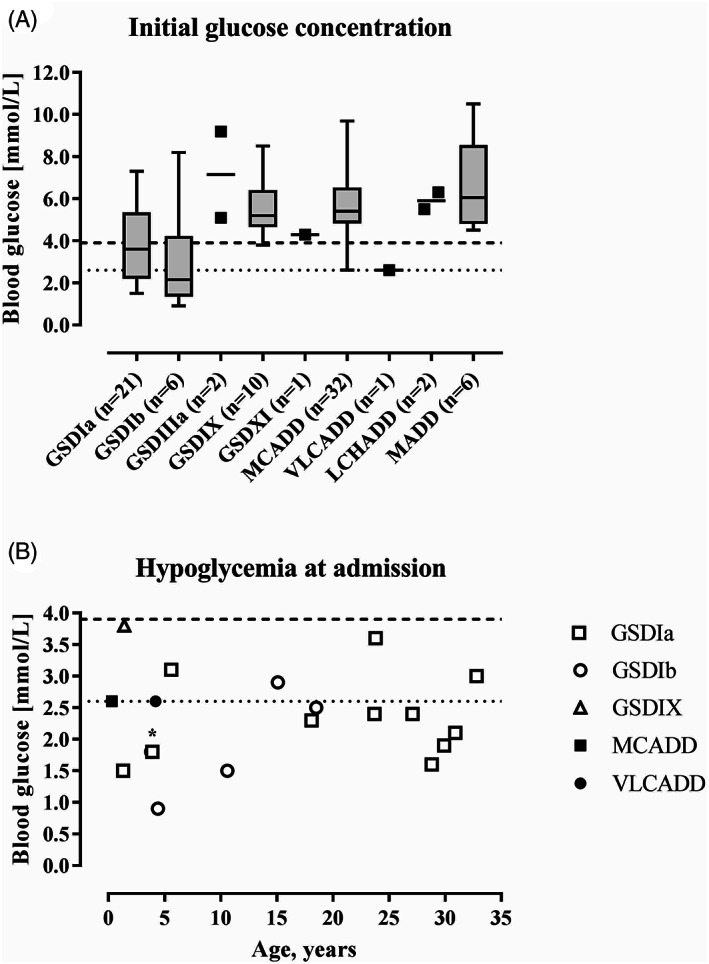
Blood glucose concentrations at hospital admission. A, Initial glucose concentrations at hospital admission per IEM (n = 81). The boxes represent the 25th to 75th percentiles, the whiskers represent the range. B, Characteristics of hypoglycemic glucose concentrations at hospital admission (n = 19). Dashed lines represent the cutoff values for hypoglycemia at 2.6 mmol/L (22) and 3.9 mmol/L (19), respectively. *; data point represents two patients with a glucose concentration of 1.8 mmol/L at the age of 4 years with GSD types Ia and Ib

Data on initial plasma glucose concentrations at admission were available for 64% of the admissions (81/127). Hypoglycemia was reported in 15% (19/127) of such admissions (Figure 2). 84% (16/19) of hypoglycemic events occurred in patients with GSDIa and Ib, and 11% (2/19) of hypoglycemic events occurred in patients with FAOD (Figure 2A). When stratifying for age, hypoglycemia was detected in all age groups in patients with GSDI, but it was uncommon in patients with FAOD aged >5 years (Figure 2B). No convulsions, coma, or death due to a metabolic decompensation were reported. One GSDIb patient died in the data collection period because of a severe dilated cardiomyopathy unrelated to metabolic decompensations. An ICU admission was documented for two patients with GSDIa, to support safe monitoring in one adult patient, and for central venous line placement in a 1‐year‐old patient. The duration of ICU treatment was 1 day in both patients and no long‐term complications due to these admissions were reported.

Lethargy was reported in patients with GSDIa (n = 2), GSDIb (n = 1), GSDXI (n = 1), MCADD (n = 4), LCHADD (n = 1), and VLCADD (n = 1). In three out of four patients with MCADD in whom lethargy was documented, glucose concentrations were above 3.9 mmol/L. No hyperammonemia was documented. Acute rhabdomyolysis was reported in two patients with LCHADD (n = 1) and VLCADD (n = 1), with maximum CK concentrations of 63 238 and 3200 U/L, respectively.

### 3.3 www.emergencyprotocol.net

The website www.emergencyprotocol.net is now freely accessible to patients and healthcare providers. The page “Leaflet” provides translated instruction leaflets on how to use the emergency protocol. The page “Emergency letter” allows the generation of personalized emergency letters. These personalized emergency letters are based on a protocol version resulting from revisions of the original UMCG generic emergency protocol, after multiple discussions and reaching final agreement on topics such as drugs and solutions calculations, laboratory tests, and aims (Figure 1).

For the generation of the emergency letters, the following basic information should be provided: patient's name, disease type, date of birth, weight, language, and primary metabolic center. To date, emergency letters can be generated for patients with the following IEMs: GSD 0, GSD Ia, GSD Ib, GSD IIIa, GSD IIIb, GSD IV, GSD VI, GSD IX, GSD XI, MCADD, VLCADD, MADD, and LCHAD/MTP deficiency. The option GSD* is added to offer a solution to modify the template for IEMs that are not listed explicitly.

The emergency letter can be generated in three different file types (ie, pdf, Word, or HTML) and currently in the following languages: Dutch, English, French, German, Greek, Italian, Polish, Portuguese, Spanish, Swedish, and Turkish.

## 4 DISCUSSION

Preventing acute metabolic decompensation by timely, prompt, and safe treatment and communication is crucially important in optimizing outcomes in IEM patients with fasting intolerance. We herein report single‐center experience with the use of a generic emergency protocol in a subgroup of IEM patients. Collected data suggest that emergency letters based on a *generic* emergency protocol can safely prevent metabolic emergencies in patients with hepatic GSD and MCADD (the most common IEM and the one for which more data were available). We also describe the development and functionality of a public website aimed at creating personalized emergency letters for patients with hepatic GSD and the main FAOD.

In the study cohort, few patients were hypoglycemic at hospital admission. Hypoglycemia was uncommon in patients with ketotic GSD and patients with FAOD aged >5 years. This is notable because an important subset of patients with IEMs has severe fasting intolerance with regular events of hypoglycemia in their daily life.^20^ Because a key objective of the study was to assess the safety of the protocols, we used a conservative definition of hypoglycemia <3.9 mmol/L (< 70 mg/dL)^21^ compared to 2.6 mmol/L as used in some other studies.^22^ Before autonomic system and neuroglycopenia‐related symptoms and signs are perceived, this threshold is commonly used in family instructions for recognizing and initiating hypoglycemia treatment. The approach taken in the emergency protocol emphasizes prevention and reversal of a catabolic state, through early intervention, timely, and relatively high carbohydrate intake (estimated based on actual body weight^17^), and prompt communication with IEM experts as needed. Meanwhile, the approach acknowledges the high level of self‐management by many IEM families. To our opinion, this combined approach likely has prevented hypoglycemias in many patients with IEMs in this study.

Convulsions, coma, or death were not reported at acute hospitalizations in the 128 patients during the 5‐year study period. Nonetheless, preventive hospital admissions were frequent among all studied IEMs. Although newborn screening for FAOD has led to a significant reduction in deaths and serious adverse events, utilization of acute care services remains high in these patients compared to age‐matched controls. In line with the present study, a retrospective cohort study in patients with IEMs identified through newborn screening between 2006 and 2007 reported that 44% (27 out of 61) of patients with an FAOD had IEM‐related acute care utilization during their first year of life.^23^ In a recent study from Canada, children with MCADD experienced on average 0.6 hospital admissions per year, between 6 and 12 months of age.^24^ Long‐term data on hospital admissions in patients diagnosed with hepatic GSD are lacking. However, an international questionnaire showed that hospital admission due to complications of dietary management occurred in 32% (79 out of 249) of patients with GSD.^20^ In the latter study, 61% of the respondents reported using an emergency letter.

Preventing catabolism and recognizing the early stages of metabolic decompensation in patients with IEM is challenging because of the IEM‐specific pathophysiology of fasting. For instance, in patients with GSDI, lactate can function as an alternative energy substrate to glucose for the brain.^25^ Consequently, overt neurologic symptoms and signs of hypoglycemia (neuroglycopenia) may be delayed in patients experiencing hypoglycemia. By contrast, in patients with FAOD hypoglycemia is a relatively late finding of metabolic decompensation and often preceded by lethargy and vomiting.^26^ Indeed, in the present study, we found that lethargy was reported in three out of four patients with MCADD in whom glucose concentrations were above the stated cutoff values for hypoglycemia. In addition, the symptoms and signs associated with fasting intolerance likely depend on the patients' ages.^12^ Although our protocol is generic, these findings underscore the importance of individualizing instructions for caregivers and patients, as the combination of education and practical and explicit clinical pathways are crucial to prevent emergency situations.^27^, ^28^ In addition, healthcare providers should be aware of the potential risks related to suboptimal emergency treatment, including electrolyte imbalance and iatrogenic hypoglycemia (if the emergency solution or glucose infusion are given late or stopped too early).

The study has some potential limitations. First, the retrospective design and the lack of interoperability and interconnectivity between different EHR systems may have introduced selection bias and information bias. For example, hospital admissions and initial laboratory studies may not always have been communicated to our center or documented in the EHR system. However, it is unlikely that metabolic decompensations causing death, coma, convulsions, and/or ICU admissions would have been missed, as the patient cohort is closely followed and shared care with the local hospitals is well organized. Second, for both organizational and ethical reasons, the study did not include a control group. Therefore, we were not able to compare the events and outcomes with, for example, a patient cohort with IEM‐specific emergency letters. Third, the study design did not allow us to assess if and to what extent starting phase I of the emergency protocol at home prevented hospital admissions (however, a higher number of [potentially unrecorded] prevented admissions would argue in favor of the protocol presented here). Conversely, delay in starting the protocol due to various reasons (eg, lack of materials at home, sociodemographic factors, patient‐related factors) might have resulted in an increased number of hospitalizations. Both early and late starting of phase I may be caused by individual patient‐related factors and should be addressed during prospective monitoring. Fourth, the study cohort included relatively few patients with FAOD other than MCADD, limiting the generalizability of the findings to all FAOD. Additionally, the study did not include patients with IEMs of the intoxication type (eg, the organic acidemia, urea cycle defects), for which outcomes after using *generic* emergency protocols remain to be assessed. Because preventing and reversing catabolism is crucially important also in intoxication type IEMs, we hypothesize that the *generic* emergency protocol that include the use of the emergency solution can be useful also in such IEMs, with the addition of further measures specific for those types of IEMs. It should also be noted that our *generic* emergency protocols, which emphasize the use of carbohydrate rich enteral or parenteral intake are contraindicated in patients on a ketogenic or carbohydrate restricted diet. Although the current version of the protocol is the result of agreement among 54 participants from 32 centers and 15 countries, the consensus could not be formally validated (eg, by Delphi methodology).

Real‐world evidence (clinical evidence derived from the analysis of real‐world data) plays an increasing role in supporting decision‐making for rare disorders. Randomized clinical trials are often not feasible, for many reasons. In rare diseases, patients are relatively few, many are children, and clinical endpoints may not have regulatory precedence. The US Food and Drug Administration (FDA) defines real‐world data as data that are routinely collected from several sources, such as EHR and disease registries. For real‐world data using EHR, data reliability and relevance are key requirements. Retrospective studies can be efficient tools to begin collecting and analyzing real‐world data. Despite several potential limitations (eg, missing elements, lack of comparability after improvements in standard‐of‐care management, referral bias), retrospective studies can be performed relatively quickly and may provide the background for longer and laborious prospective studies.^29^

We have previously digitalized the emergency protocol as part of the GSD Communication Platform, a telemedicine platform for patients with hepatic GSD.^30^ The website www.emergencyprotocol.net supports a shared care model, which uses the medical and communication competences of all stakeholders: the metabolic center of expertise, the local healthcare providers, the caregivers, and the patients, who all share joint responsibility. In this respect, the emergency protocol does not to replace expert metabolic advice; the connection with the responsible metabolic center remains an important step in patients' management. These emergency letters and the website can help to focus decision taking. However, emergency letters, clinical care pathways, and evidence‐based guidelines can never replace clinical expertise when making treatment decisions for individual patients. The doctor‐patient relationship needs to guarantee that personal values, preferences, and individual circumstances (including psychosocial and cultural aspects) are taken into account. For these reasons, next steps may include a value‐based healthcare process toward personalized medicine, by implementing patient's perspectives, to strike the most effective balance between timely management and avoiding overtreatment. Since www.emergencyprotocol.net is constantly updated as part of a continuous process, future discussion, revision, and validation within the IEM (professional and patient) community are also expected to lead to further improvements of the emergency letters.

## 5 CONCLUSION

A *generic* emergency protocol can be safe for home management by caregivers and for initial (first hour) in‐hospital management of metabolic emergencies in patients with hepatic GSD and MCADD. Even though IEM‐specific emergency letters are widely used, a simple generic emergency protocol, which can be generated online at any time, can be easier to use for families and local physicians before contacting the metabolic specialist. Disseminating such emergency protocol methods and assessing outcomes are crucial next steps aimed at improving further care and prevention, developing an international consensus among healthcare providers, and fostering prospective research studies in patients with IEMs.

## CONFLICT OF INTEREST

The authors declare no potential conflict of interest.

## MEMBERS OF THE CONNECT MetabERN COLLABORATION GROUP

**Lut de Baere**, Belgische Organisatie voor Kinderen en volwassenen met een Stofwisselingsziekte VZW, Belgium.

**Cinzia Bellettato**, MetabERN, Regional Coordinating Center for Rare Diseases, Udine University Hospital, Udine, Italy.

**Annet M Bosch**, Department of Pediatrics, Emma Children's Hospital, Amsterdam Gastroenterology, Endocrinology & Metabolism, Amsterdam UMC, University of Amsterdam, Amsterdam, The Netherlands.

**Julieta Bonvin Sallago**, Department of Pediatrics, University of Connecticut School of Medicine, Connecticut Children's Hospital, Hartford, Connecticut, USA.

**Lorenzo D. Botto**, Division of Medical Genetics, Department of Pediatrics, University of Utah, Salt Lake City, Utah, USA.

**Michaela Brunner‐Krainz**, Department of Pediatrics, Medical University of Graz, Graz, Austria.

**Camilla Carøe**, Paediatric Nutrition, University Hospital of Copenhagen, Rigshospitalet, Copenhagen, Denmark.

**Thomas Casswall**, Department of Pediatric Gastroenterology, Hepatology and Nutrition, Karolinska University Hospital, Stockholm, Sweden.

**Enrique Landelino Contreras Pulido,** Patient Representative on behalf of the Spanish Association of GSD Patients ‐ AEEG.

**Maria L. Couce**, Complejo Hospitalario Universitario de Santiago de Compostela, Spain.

**Anne‐Frederique Dessein**, University Hospital of Lille, France.

**Maria Alice Donati**, Department of Inherited Neuro‐metabolic Disorders, Anna Meyer Children's Hospital, Florence, Italy.

**Francois Eyskens**, Center of Metabolic Diseases, University Hospital, Antwerp, Belgium.

**Carolina Fischinger Moura De Souza**, Hospital de Clínicas de Porto Alegre (HCPA), Serviço de Genética Médica, Porto Alegre, Brazil.

**Pilar Quijada Fraile**, Pediatric Department, University Hospital 12 de Octubre, Madrid, Spain.

**Sabine Annemijn Fuchs**, Department of Metabolic Diseases, Wilhelmina Children's Hospital, University Medical Center Utrecht, Utrecht, The Netherlands.

**Serena Gasperini**, Rare Metabolic Diseases Pediatric Center, Pediatric Clinic, Fondazione MBBM, San Gerardo Hospital, Monza, Italy.

**Dorothea Haas**, Department of Pediatrics, Centre for Pediatric and Adolescent Medicine, Division of Neuropaediatrics and Metabolic Medicine, University Hospital, Heidelberg, Germany.

**Elena Martín Hernández**, Unit of Mitochondrial and Inherited Metabolic Diseases, Pediatric Department, University Hospital 12 de Octubre, Madrid, Spain.

**Michel Hochuli**, Department of Diabetes, Endocrinology, Nutritional Medicine and Metabolism, Inselspital, Bern University Hospital and University of Bern, Bern, Switzerland.

**Anne Hugon**, Association Francophone des Glycogènoses, France.

**Daniela Karall**, Clinic of Pediatrics, Division of Inherited Metabolic Disorders, Medical University of Innsbruck, Innsbruck, Austria.

**Dwight Koeberl**, Division of Medical Genetics, Department of Pediatrics, Duke University Medical Center, Durham, USA.

**Philippe Labrune**, Reference Center for Inherited Metabolic Diseases, Antoine Béclère Hospital, APHP, Filière G2M, MetabERN, Clamart, France.

**Svetlana Lajic**, Pediatric Endocrinology Unit, Astrid Lindgren Children's Hospital, Karolinska University Hospital, Stockholm, Sweden.

**Corine van Lingen**, MetabERN, Regional Coordinating Center for Rare Diseases, Udine University Hospital, Udine, Italy.

**Arianna Maiorana**, Division of Metabolism, Bambino Gesù Children's Hospital, IRCCS, Rome, Italy.

**Karine Mention**, University Hospital of Lille, France.

**Isabelle Moenig**, Department of Pediatrics, Schleswig‐Holstein University Hospital, Lübeck, Germany.

**Klaus Mohnike**, Department of Paediatrics, Otto‐von‐Guericke University, Magdeburg, Germany.

**Chiara Montanari**, Department of Pediatrics, San Paolo Hospital, ASST Santi Paolo e Carlo, University of Milan, Milan, Italy.

**Marie‐Cécile Nassogne**, Cliniques universitaires St Luc (Bruxelles)‐Université catholique de Louvain, Belgium.

**Rossella Parini**, Rare Metabolic Diseases Adult Center, San Gerardo Hospital, Monza, Italy.

**Shamima Rahman**, Metabolic Unit, Great Ormond Street Hospital for Children NHS Foundation Trust, London, UK.

**Magali Reyes**, Hospital Infantil de México Federico Gómez, Mexico City, Mexico.

**Marit Schwantje**, Department of Metabolic Diseases, Wilhelmina Children's Hospital, University Medical Center Utrecht, Utrecht, The Netherlands.

**Anastasia Skouma**, Institute of Child Health, Agia Sofia Children's Hospital, Athens, Greece.

**Pietro Strisciuglio**, Department of Translational Medicine, Section of Pediatrics, University of Naples “Federico II", Naples, Italy.

**Maren Thiel**, German speaking self‐help group for inborn fatty acid oxidation disorders ‐ Fett‐SOS e.V., Germany.

**David Weinstein**, Connecticut Children's Hospital, Hartford, Connecticut, USA.

**Athanasia Ziagaki**, Interdisciplinary Center of Metabolism: Endocrinology, Diabetes and Metabolism, University‐Medicine Berlin, Berlin Germany.

## References

[1] Grunewald S , Davison J , Martinelli D , Duran M , Dionisi‐Vici C . Emergency diagnostic procedures and emergency treatment. In: Blau N , Duran M , Gibson K , Dionisi‐Vici C , eds. Physician's Guide to the Diagnosis, Treatment, and Follow‐Up of Inherited Metabolic Diseases. Berlin Heidelberg: Springer‐Verlag; 2014:709.

[2] Dixon MA , Leonard JV . Intercurrent illness in inborn errors of intermediary metabolism. Arch Dis Child. 1992;67(11):1387‐1391. 1471895 10.1136/adc.67.11.1387 PMC1793747

[3] Prietsch V , Lindner M , Zschocke J , Nyhan W , Hoffmann G . Emergency management of inherited metabolic disorders. J Inherit Metab Dis. 2002;25:531‐546. 12638937 10.1023/a:1022040422590

[4] Van Hove JLK , Myers S , Vande KK , Freehauf C , Bernstein L . Acute nutrition management in the prevention of metabolic illness: a practical approach with glucose polymers. Mol Genet Metab. 2009;97(1):1‐3. 19367873 10.1016/j.ymgme.2009.03.001

[5] Rodan LH , Aldubayan SH , Berry GT , Levy HL . Acute illness protocol for urea cycle disorders. Pediatr Emerg Care. 2018;34(6):e115‐e119. 29135898 10.1097/PEC.0000000000001298

[6] Rodan LH , Aldubayan SH , Berry GT , Levy HL . Acute illness protocol for maple syrup urine disease. Pediatr Emerg Care. 2018;34(1):64‐67. 29095391 10.1097/PEC.0000000000001299

[7] Aldubayan SH , Rodan LH , Berry GT , Levy HL . Acute illness protocol for organic acidemias: methylmalonic acidemia and propionic acidemia. Pediatr Emerg Care. 2017;33(2):142‐146. 28141776 10.1097/PEC.0000000000001028

[8] Aldubayan SH , Rodan LH , Berry GT , Levy HL . Acute illness protocol for fatty acid oxidation and carnitine disorders. Pediatr Emerg Care. 2017;33(4):296‐301. 28353532 10.1097/PEC.0000000000001093

[9] Kölker S , Christensen E , Leonard JV , et al. Guideline for the diagnosis and management of glutaryl‐CoA dehydrogenase deficiency (glutaric aciduria type I). J Inherit Metab Dis. 2007;30(1):5‐22. 17203377 10.1007/s10545-006-0451-4

[10] Rake JP , Visser G , Labrune P , Leonard JV , Ullrich KS . Guidelines for management of glycogen storage disease type I—European study on glycogen storage disease type I ( ESGSD I ). Eur J Pediatr. 2002;161:S112‐S119. 12373584 10.1007/s00431-002-1016-7

[11] Visser G , Rake J , Labrune P , et al. Consensus guidelines for management of glycogen storage disease type 1b—European study on glycogen storage disease type 1. Eur J Pediatr. 2003;161:S120‐S123. 10.1007/s00431-002-1017-6 12373585

[12] Kishnani PS , Austin SL , Arn P , et al. Glycogen storage disease type III diagnosis and management guidelines. Genet Med. 2010;12:446‐463. 20631546 10.1097/GIM.0b013e3181e655b6

[13] Kishnani PS , Austin SL , Abdenur JE , et al. Diagnosis and management of glycogen storage disease type I: a practice guideline of the American College of Medical Genetics and Genomics. Genet Med. 2014;128:1‐29. 10.1038/gim.2014.128 25356975

[14] Kishnani PS , Goldstein J , Austin SL , et al. Diagnosis and management of glycogen storage diseases type VI and IX: a clinical practice resource of the American College of Medical Genetics and Genomics (ACMG). Genet Med. 2019;21(4):772‐789. 30659246 10.1038/s41436-018-0364-2

[15] Peeks F , Boonstra W , de Baere L , et al. Research priorities for liver glycogen storage disease: an international priority setting partnership with the James Lind Alliance. J Inherit Metab Dis. 2020;43(2):279‐289. 31587328 10.1002/jimd.12178 PMC7079148

[16] Hawkes CP , Walsh A , O'Sullivan S , Crushell E . Doctors' knowledge of the acute management of inborn errors of metabolism. Acta Paediatr Int J Paediatr. 2011;100(3):461‐463. 10.1111/j.1651-2227.2010.02062.x 21133998

[17] Bier DM , Leake RD , Haymond MW , et al. Measurement of “true” glucose production rate in infancy and childhood with 6,6‐dideuteroglucose. Diabetes. 1977;26(11):1016‐1023. 913891 10.2337/diab.26.11.1016

[18] Harris PA , Taylor R , Thielke R , Payne J , Gonzalez N , Conde JG . Research electronic data capture (REDCap)‐a metadata‐driven methodology and workflow process for providing translational research informatics support. J Biomed Inform. 2009;42(2):377‐381. 18929686 10.1016/j.jbi.2008.08.010 PMC2700030

[19] Schwartz NS , Clutter WE , Shah SD , Cryer PE . Glycemic thresholds for activation of glucose counterregulatory systems are higher than the threshold for symptoms. J Clin Invest. 1987;79(3):777‐781. 3546378 10.1172/JCI112884 PMC424197

[20] Steunenberg TAH , Peeks F , Hoogeveen IJ , et al. Safety issues associated with dietary management in patients with hepatic glycogen storage disease. Mol Genet Metab. 2018;125:79‐85. 30037503 10.1016/j.ymgme.2018.07.004

[21] American Diabetes Association . Defining and reporting hypoglycemia in diabetes. A report from the American Diabetes Association Workgroup on Hypoglycemia. Diabetes Care. 2005;28:1245‐1249. 15855602 10.2337/diacare.28.5.1245

[22] Koh THHG , Aynsley‐Green A , Tarbit M , Eyre JA . Neural dysfunction during hypoglycaemia. Arch Dis Child. 1988;63(11):1353‐1358. 3202642 10.1136/adc.63.11.1353 PMC1779138

[23] Wang Y , Sango‐Jordan M , Caggana M . Acute care utilization for inherited metabolic diseases among children identified through newborn screening in New York state. Genet Med. 2014;16(9):665‐670. 24625447 10.1038/gim.2014.21

[24] Karaceper MD , Khangura SD , Wilson K , et al. Health services use among children diagnosed with medium‐chain acyl‐CoA dehydrogenase deficiency through newborn screening: a cohort study in Ontario, Canada. Orphanet J Rare Dis. 2019;14(1):4‐13. 30902101 10.1186/s13023-019-1001-0 PMC6431026

[25] Fernandes J , Berger R , Smit GPA . Lactate as energy source for brain in glucose‐6‐phosphatase deficient child. Lancet. 1982;319(8263):113. 10.1016/s0140-6736(82)90257-4 6119484

[26] Morris AAM , Leonard JV . Early recognition of metabolic decompensation. Arch Dis Child. 1997;76(6):555‐556. 9245861 10.1136/adc.76.6.555 PMC1717221

[27] Zand DJ , Brown KM , Lichter‐Konecki U , Campbell JK , Salehi V , Chamberlain JM . Effectiveness of a clinical pathway for the emergency treatment of patients with inborn errors of metabolism. Pediatrics. 2008;122(6):1191‐1195. 19047233 10.1542/peds.2008-0205

[28] Wilson CJ , Champion MP , Collins JE , Clayton PT , Leonard JV . Outcome of medium chain acyl‐CoA dehydrogenase deficiency after diagnosis. Arch Dis Child. 1999;80(5):459‐462. 10208954 10.1136/adc.80.5.459 PMC1717923

[29] Wu J , Wang C , Toh S , Pisa FE , Bauer L . Use of real‐world evidence in regulatory decisions for rare diseases in the United States‐current status and future directions. Pharmacoepidemiol Drug Saf. 2020;29(10):1213‐1218. 32003065 10.1002/pds.4962

[30] Hoogeveen IJ , Peeks F , de Boer F , et al. A preliminary study of telemedicine for patients with hepatic glycogen storage disease and their healthcare providers: from bedside to home site monitoring. J Inherit Metab Dis. 2018;41(6):929‐936. 29600495 10.1007/s10545-018-0167-2 PMC6326981

